# Blocking 5-HT2 receptor restores cardiovascular disorders in type 1 experimental diabetes

**DOI:** 10.1038/srep33979

**Published:** 2016-09-23

**Authors:** José-Ángel García-Pedraza, Pedro Ferreira-Santos, Rubén Aparicio, María-José Montero, Asunción Morán

**Affiliations:** 1 Laboratory of Pharmacology, Department of Physiology and Pharmacology, Faculty of Pharmacy, University of Salamanca, 37007, Salamanca, Spain; 2 Biomedical Research Institute of Salamanca (IBSAL), University Hospital of Salamanca-USAL- CSIC, Salamanca, Spain

## Abstract

This study aimed to determine whether the serotonergic modulation, through selective 5-HT_2_ receptor blockade, restores cardiovascular disturbances in type 1 diabetic rats. Diabetes was induced by alloxan (150 mg/kg, s.c.) and maintained for 4 weeks. 5-HT_2_ receptor was blocked by sarpogrelate (30 mg/kg.day; 14 days; p.o.). Systolic blood pressure (SBP), heart rate (HR), glycaemia and body weight (BW) were monitored periodically. Animals were sacrificed at the end of the study and the heart, right kidney and thoracic aorta were removed; plasma samples were also obtained. Left ventricular hypertrophy index (LVH) and renal hypertrophy index (RH) were determined. Vascular function was studied in aorta rings; additionally, superoxide anion (O_2_•^−^) production (by lucigenin-enhanced chemiluminescence) and lipid peroxidation (by thiobarbituric acid reactive substances assay) were measured. Neither alloxan nor sarpogrelate treatments altered HR, LVH or endothelium-independent relaxation. SBP, glycaemia, BW, RH, O_2_•^−^ production and lipid peroxidation were significantly altered in diabetic animals compared with controls. Sarpogrelate treatment considerably decreased SBP, RH, O_2_•^−^ production and lipid peroxidation. Endothelium-dependent relaxation was severely reduced in diabetic animal aortas compared to controls; sarpogrelate treatment markedly improved it. Our outcomes show that selectively blocking 5-HT_2_ receptors has beneficial effects on impaired cardiovascular parameters in diabetes.

Endothelial dysfunction plays a fundamental role in the pathophysiology of diabetes-induced cardiovascular complications, which remain the leading cause of morbidity and mortality in patients with type 1 diabetes (T1D). T1D is a severe and chronic disease characterized by a complete insulin deficiency ending with an extremely high concentration of blood glucose; the hyperglycaemia, as hallmark of diabetes, is involved in the pathogenesis of endothelial dysfunction, which precedes both micro- and macrovascular complications of diabetes^1^,^2^,^3^.

Although insulin therapy attempts to restore normal blood glucose values, it has been shown that even an optimal glycaemic control do not fully protect against, fix or target the cardiovascular complications occurring during T1D^4^. Therefore, depth knowledge in the mechanisms of cardiovascular diseases and novel approaches to treat cardio and vasculopathies is extremely crucial^4^,^5^,^6^. In this sense, the serotonergic system stands out for its relevance in the diabetic pathophysiology, since: (i) 5-HT concentrations are altered in diabetes^7^,^8^; (ii) 5-HT inhibits the peripheral sympathetic neurotransmission in type 1 diabetic rats^9^,^10^; (iii) it has been described an increase in serotonergic peripheral actions, mainly by 5-HT_2_ receptor activation (increasing platelet aggregation or contractile responses)^11^,^12^,^13^,^14^,^15^ and (iv) 5-HT_2_ receptor activation is involved in an enhanced serotonergic vasoconstriction in the type 1 diabetic rat kidney^16^. Taking into consideration the above-mentioned evidence, 5-HT_2_ receptor seems to trigger harmful actions at cardiovascular level (whose actions are amplified in T1D). Thus, several investigations have demonstrated that selective 5-HT_2_ blockade displays protective effects in both T1D and type 2 diabetes^17^,^18^,^19^,^20^,^21^; in this study, we aim to determine the impact of modulating the serotonergic system, by the selective blockade of the 5-HT_2_ receptors (sarpogrelate), on the development of hypertension, cardiac and renal hypertrophy, oxidative stress and endothelial dysfunction in an experimental model of T1D. The rational of our study is based on recent data where our group showed that orally chronic treatment with a selective 5-HT_2_ antagonist (sarpogrelate; 30 mg/kg.day) exerted cardiovascular favourable actions by enhancing the 5-HT inhibition of the sympathetic neurotransmission^22^,^23^, and exhibiting 5-HT vasodilation induced by nitric oxide (NO), cyclooxygenase (COX) pathway and K^+^-ATP channels in the rat renal bed^24^. We believe that by studying the impact of the serotonergic system in diabetes we will shed a light to a possible therapeutic target in cardiovascular complications because of chronic hyperglycaemia.

## Results

### Blood glucose, body weight, heart rate and systolic blood pressure measurements

Alloxan administration elicited a marked increase in blood glucose concentration and decreased body weight (BW) when compared to the normoglycaemic (control) rats. Sarpogrelate treatment did not alter either the hyperglycaemia or the BW when compared with diabetic group (Table 1).

After 28 days of the induction of diabetes the animals reached a hypertensive state (see Table 1), which was mitigated in the group of diabetic rats treated with sarpogrelate. However, heart rate (HR) was not modified either with alloxan or with sarpogrelate treatment when compared to control rats (Table 1).

### Cardiac and renal hypertrophy

**Figure 1:**
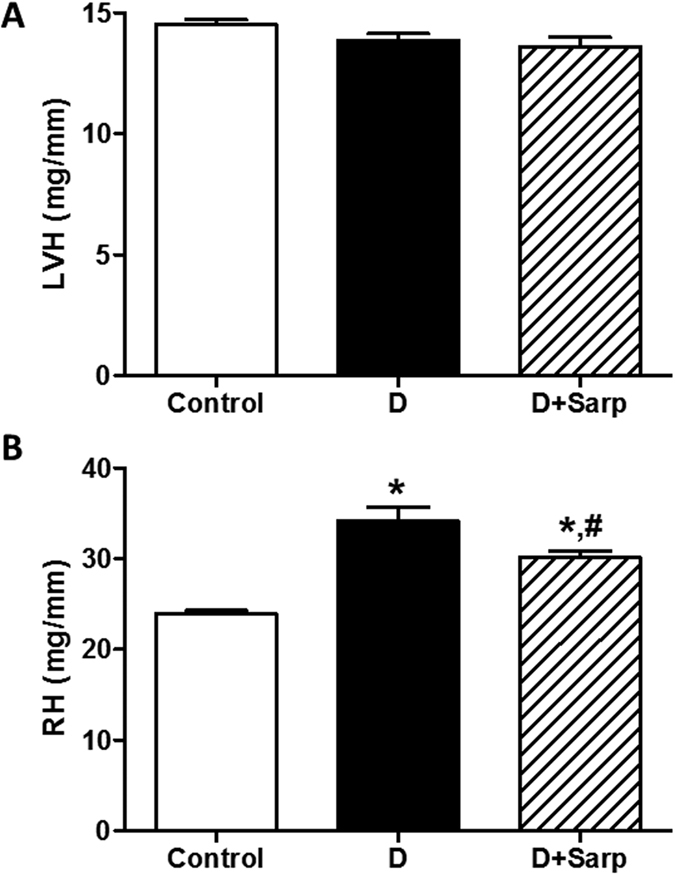
Cardiac and renal hypertrophy. Relation between the weight of the left ventricle (**A**) or the weight of kidney (**B**) and the tibia length, used as left ventricular hypertrophy index (LVH) or renal hypertrophy index (RH), respectively, in normoglycaemic group (Control), diabetic group (**D**) and sarpogrelate-treated diabetic group (D+Sarp). Values are expressed as mean ± SEM (n = 5–8). *P < 0.05 *vs* control group. ^#^P < 0.05 *vs* diabetic group.

The left ventricle hypertrophy (LVH) index was not different among all the studied groups (Fig. 1A). However, the renal hypertrophy (RH) index was significantly enhanced in diabetic group *vs* control group; sarpogrelate treatment was capable of markedly reducing this index (Fig. 1B).

### Aortic contractile responses to phenylephrine

The contractile response to phenylephrine (PE; 10^−6^ M) in aortic rings was 1756.0 ± 48.4 mg in control rats; this contraction was significantly higher in non-treated diabetic group, 2225.0 ± 101.2 mg (P < 0.05 *vs* control rats). Sarpogrelate treatment was able to reduce this increased contractile response in diabetic rats, to the same level as the control group (1862.0 ± 66.9 mg) (P > 0.05 *vs* control rats) (n = 8 each group).

### Endothelium-dependent relaxation in aorta rings

**Figure 2:**
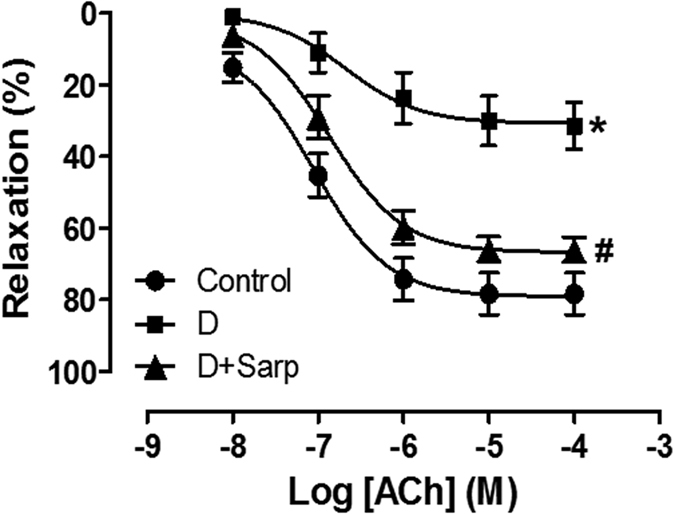
Aortic endothelium-dependent relaxation. Relaxation induced by acetylcholine (ACh) in aorta from normoglycaemic group (Control), diabetic group (D) and sarpogrelate-treated diabetic group (D+Sarp). Values are expressed as mean ± SEM (n = 8 each). *P < 0.05 *vs* control group. ^#^P < 0.05 *vs* diabetic group.

Aortic rings from diabetic rats showed decreased endothelium-dependent vasodilator responses to acetylcholine (ACh), when compared to aortas from normoglycaemic rats. Sarpogrelate treatment produced a significant increase in the relaxation induced by ACh, compared with non-treated diabetic rats (Fig. 2).

**Figure 3:**
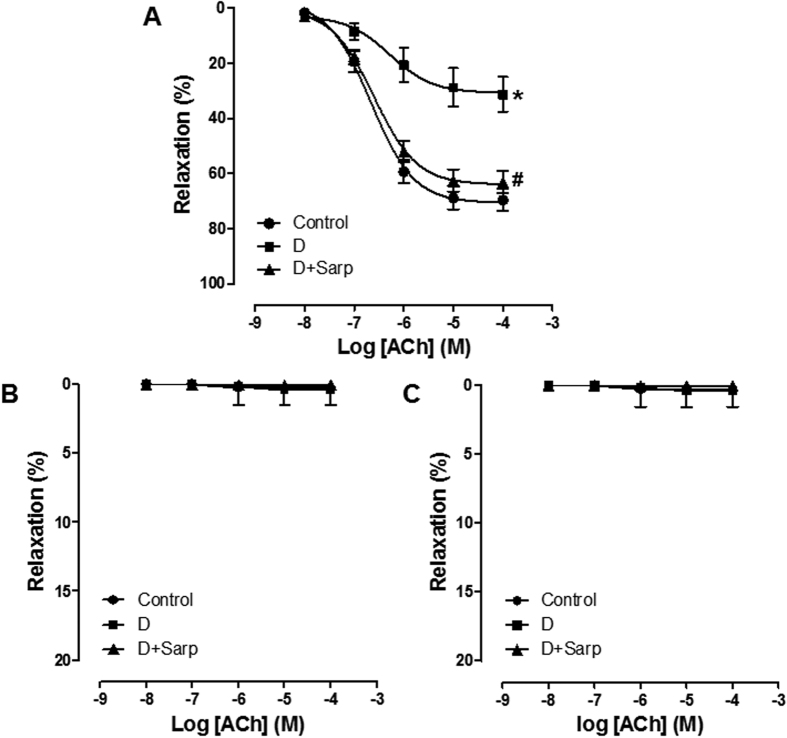
NO, COX and endothelium-derived hyperpolarization pathways in aortic endothelium-dependent relaxation. Concentration–response curves to acetylcholine (ACh) in the presence of: (**A**) indomethacin (10^−5^ M) plus TEA (10^−4^ M), (**B**) L-NAME (10^−4^ M) plus TEA, or (**C**) L-NAME plus indomethacin in aorta from normoglycaemic group (Control), diabetic group (**D**) and sarpogrelate-treated diabetic group (D+Sarp). Values are expressed as mean ± SEM (n = 3–5). *P < 0.05 *vs* control group. ^#^P < 0.05 *vs* diabetic group.

After incubation with the combination of indomethacin and tetraethylammonium (TEA), the NO-mediated relaxation was significantly smaller in preparations of diabetic compared to normoglycaemic and sarpogrelate-treated diabetic rats (Fig. 3A). Nevertheless, in the presence of Nω-Nitro-L-arginine methyl ester (L-NAME) plus TEA (COX-mediated relaxation) (Fig. 3B) or L-NAME plus indomethacin (endothelium-dependent hyperpolarization-type relaxation) (Fig. 3C) the ACh response was abolished in all study groups.

### Endothelium-independent relaxation in aorta rings

**Figure 4:**
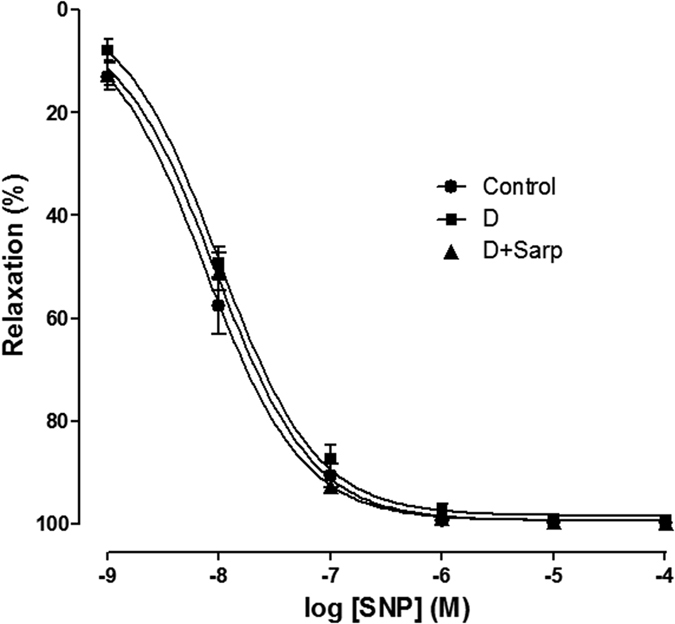
Aortic endothelium-independent relaxation. Vascular relaxation to sodium nitroprusside (SNP) in aorta from normoglycaemic group (Control), diabetic group (D) and sarpogrelate-treated diabetic group (D+Sarp). Values are expressed as mean ± SEM (n = 4–7).

The relaxation induced by sodium nitroprusside (SNP) reached 100% in all groups. No difference was observed in the sensitivity of the aortic arteries to SNP in any of the studied groups (Fig. 4).

### Oxidative stress determination

**Figure 5:**
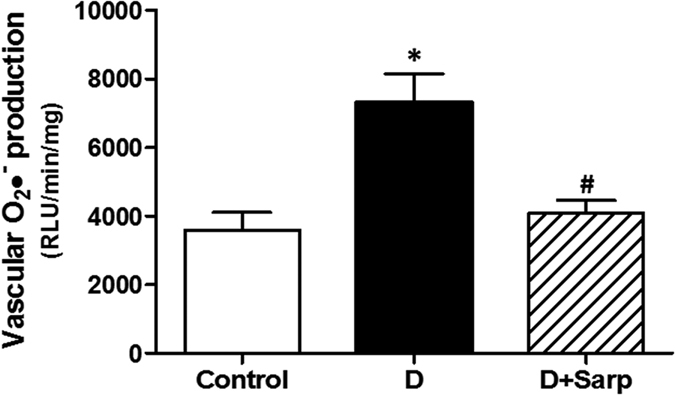
Superoxide anion determination. Vascular superoxide anion (O_2_•^−^) level expressed as relative luminescence units (RLU)/min/mg dry tissue stimulated by NADPH addition in aortic rings from normoglycaemic group (Control), diabetic group (D) and sarpogrelate-treated diabetic group (D+Sarp). Values are expressed as mean ± SEM (n = 6). *P < 0.05 *vs* control group. ^#^P < 0.05 *vs* diabetic group.

Figure 5 shows the superoxide anion (O_2_•^−^) production, stimulated by nicotinamide adenosine dinucleotide phosphate (NADPH), in aortic rings in all experimental groups. Alloxan-induced diabetes caused a significant increase in the concentration of this free radical. Interestingly, sarpogrelate treatment decreased the amount of O_2_•^−^, to the same level as the control group.

**Figure 6:**
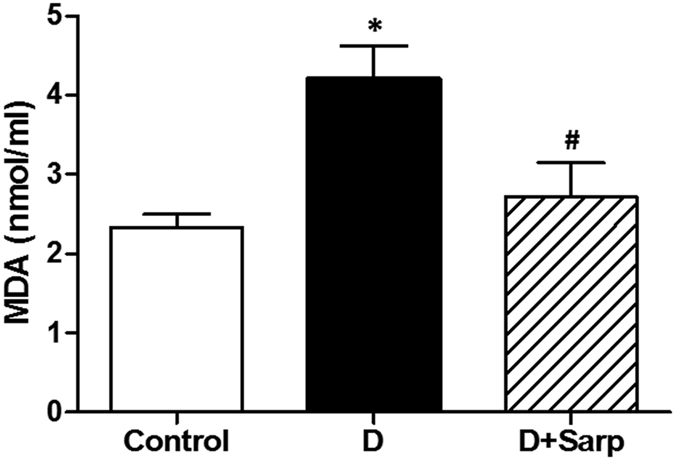
Lipid peroxidation determination. Plasmatic malondialdehyde (MDA) concentration (nmol/ml) from normoglycaemic group (Control), diabetic group (D) and sarpogrelate-treated diabetic group (D+Sarp). Values are expressed as mean ± SEM (n = 4–7). *P < 0.05 *vs* control group. ^#^P < 0.05 *vs* diabetic group.

Similarly, lipid peroxidation, determining the plasmatic malondialdehyde (MDA) levels, significantly increased in non-treated diabetic group (Fig. 6); nonetheless, sarpogrelate treatment was able to strongly reduce this deleterious effect.

## Discussion

As outlined in the Introduction section, the goal of the present study was to determine whether the modulation of 5-HT system through the selective blockade of 5-HT_2_ receptors (sarpogrelate) could rescue the damaged cardiovascular parameters in T1D. To accomplish our aim, we used alloxan-induced type 1 diabetic rat model characterized by a specific necrosis of the pancreatic beta cells ending in insulinopenia and pathologically elevated blood glucose levels^9^,^10^,^11^,^16^,^25^,^26^.

Serotonergic system plays an important role in the pathophysiology of several cardiovascular disturbances; regarding diabetes, it has been shown that both its concentrations and its receptors are altered, contributing to endothelial damage and, consequently, to the onset or worsening of cardiovascular complications resulting from diabetes^7^,^8^,^27^,^28^. It has been already demonstrated that alloxan-induced T1D in rats modified the serotonergic influence on peripheral sympathetic and cholinergic neurotransmission^9^,^10^,^26^, as well as on the renal vasculature tone enhancing the 5-HT vasoconstrictor responses mediated by 5-HT_2A_^16^. Some investigations have already proven that activation of 5-HT_2_ receptors play a crucial role in the vasoconstrictor actions and platelet aggregation, evidencing that the antagonism of these serotonergic receptors could exert beneficial actions at cardiovascular level^15^,^17^,^18^,^29^,^30^. Sarpogrelate, a selective 5-HT_2_ receptor antagonist, is used in patients with arteriosclerosis obliterans to improve ischemic symptoms such as ulcer, pain and coldness of the extremities relate to chronic arterial occlusion^17^,^29^. However, this serotonergic blocker has shown multiple benefits in a great variety of cardiovascular diseases, hence it is attributed pleiotropic properties that trigger such therapeutic effects^17^,^18^. Thereby, our research team has demonstrated that sarpogrelate treatment in normoglycaemic rats caused: (i) a potentiation of peripheral sympatho-inhibition by serotonergic system^22^,^23^, and (ii) exhibited to 5-HT as an exclusively vasodilator agent in the kidney^24^, confirming that the 5-HT_2_ blockade seems to have positive effects on cardiovascular level.

Cardiovascular risk in T1D associated with hypertension is substantially enhanced^2^,^31^. In fact, chronic hyperglycaemia can trigger hypertension and, therefore, an increase in damage on the target organs (blood vessels, kidney and retina, among others). In our experimental model, T1D for 28-days duration reached an incipient hypertensive state (without modifying heart rate) compared with normoglycaemic rats; interestingly, sarpogrelate treatment significantly reduced the increase in the systolic blood pressure (SBP). Although the beneficial effect of sarpogrelate treatment on the cardiovascular system has been well documented, its antihypertensive effect has not been reported elsewhere^17^,^18^,^32^; nevertheless, our findings show that blocking 5-HT_2_ receptors during 14 days with sarpogrelate exerts a protective effect on the onset of hypertension in T1D.

In order to assess the early onset of diabetic cardiomyopathy and nephropathy, we study LVH and RH, respectively. Substantial changes were not observed in LVH in the diabetic groups compared to control animals, which can be probably attributed to the fact that the duration of diabetes is not sufficient to trigger hypertrophy of the left ventricle. However, RH was significantly increased in non-treated diabetic rats, which may be related to an incipient diabetic nephropathy; sarpogrelate treatment was able to reverse RH in diabetic rats. This result is in agreement with several studies showing that sarpogrelate offers beneficial actions in the renal pathology derived from diabetes^19^,^33^,^34^,^35^; additionally, previous data by us^24^ demonstrated that sarpogrelate is able to exhibit exclusively the vasodilator serotonergic action in the rat kidney, which may also contribute to ameliorate the renal function. The rescue of the kidney hypertrophy by sarpogrelate treatment could help to the reduction of the hypertension state and, as a whole, the onset of diabetic nephropathy.

On the other hand, neither hyperglycaemia nor BW evolution were modified by sarpogrelate treatment in type 1 diabetic rats. Conversely, other authors, in different experimental models of type 2 diabetes, have established that oral sarpogrelate treatment did improve glycaemia of diabetic rats^36^,^37^. Thus, we suggest that pharmacological benefits observed in diabetic rats treated with sarpogrelate are not due to the improvement of blood glucose levels in our experimental model.

Diabetes-triggered alterations in vascular reactivity in a conduit vessel such as the aorta have an important pathophysiological relevance because of modifications in blood flow to the heart and/or more distal peripheral blood vessels^38^. In our experimental conditions, the endothelial function in diabetic rats was deteriorated as shown by the considerable decrease in the endothelial-dependent relaxation to ACh and the increase in the vasoconstriction in response to PE. These results are consistent with other studies where both features appeared in T1D^4^,^39^; our results show that sarpogrelate treatment restored both endothelium-dependent relaxation and vasoconstrictor responses in diabetic animals.

It has been established that diabetes is associated with adrenergic hyperactivity leading to worsening of cardiovascular disorders^40^ which can explain the increase in the vasoconstrictor responses shown in our diabetic rats; on the other hand, previous studies have already demonstrated that sarpogrelate treatment potentiated the serotonergic inhibition on the peripheral sympathetic neurotransmission^22^,^23^, being in agreement with our current data where sarpogrelate treatment stops such sympathetic overactivity in diabetic rats.

Although this impaired endothelial function is mainly characterized by decreased release of NO^39^,^41^, COX-derived prostaglandins or endothelium-dependent hyperpolarization also play an important role in the endothelium-dependent relaxation^42^,^43^,^44^. However, our data indicate that endothelium-dependent relaxation mediated by COX pathway or endothelium-dependent hyperpolarization are not involved in the vasodilator effect of our three experimental groups. NO pathway was the main implicated in the endothelium-dependent relaxation and was strikingly impaired in diabetic rats; interestingly, sarpogrelate treatment potentiated the NO pathway, improving significantly the vasodilator action, in diabetic rats. These outcomes are consistent with other studies that establish that 5-HT_2_ blockade improved the role of NO in diabetic mice^45^, and in non-diabetic canine^46^, rat^47^, rabbit^48^ or guinea-pig^49^, and with previous studies where sarpogrelate treatment enhanced endothelial NO synthase expression in rats^24^.

Semaming *et al*.^50^ have already established that the endothelium-independent relaxation by SNP was damage in streptozotocin-induced T1D (6-weeks duration) in Sprague-Dawley rats, however our T1D model demonstrates that endothelial-independent relaxation induced by SNP was not affected either by alloxan administration or by treatment with sarpogrelate, which allows us to rule out an alteration on the vascular smooth muscle. What is more, these results are in agreement with several authors reporting no impairment in endothelium-independent relaxation^6^,^51^,^52^.

On this basis, these findings confirm that blocking selectively 5-HT_2_ serotonergic receptor improves endothelial function throughout NO bioavailability in alloxan-induced T1D.

Strong evidences point out an intimate association between long-time hyperglycaemia and overproduction of reactive oxygen species (ROS), which significantly contribute to the generation of both micro- and macrovascular diabetes complications^53^. In this sense, our T1D model was associated with a significant increase in both O_2_•^−^ production in aortic arteries and lipid peroxidation compared to normoglycaemic rats. Interestingly, sarpogrelate treatment was able to considerably reduce the ROS as well as the plasma levels of MDA in alloxan-induced T1D. These findings are consistent with other studies where sarpogrelate demonstrated the ability to attenuate oxidative stress^20^,^54^. Thus, taking into account these results, we could state that sarpogrelate has antioxidant properties, decreasing hyperglycaemia-induced oxidative stress. Therefore, sarpogrelate could occupy an important place in the new pharmacological approach moderating the levels of free radicals, as already reported for other compounds such as antioxidants, statins or angiotensin-converting enzyme inhibitors^55^,^56^.

Given that: (i) diabetic vasculopathies are the leading causes of morbidity and mortality in T1D^57^; (ii) despite having a suitable glycaemic control, this is not sufficient for the prevention and treatment of cardiovascular pathologies resulting from diabetes, (iii) our current outcomes exhibit that blocking selectively 5-HT_2_ receptors in T1D rats improves not only the endothelial function but also vascular abnormalities produced by chronic hyperglycaemia (development of hypertension and increased kidney hypertrophy, O_2_•^−^ production and lipid peroxidation), (iv) it has been reported that 5-HT_2_ receptor antagonists are more effective than COX inhibitors in preventing cardiovascular events in diabetic patients^58^, and (v) it has been established that sarpogrelate exerts pleiotropic effect on the vasculature resulting in the improvement of endothelial function in diabetic angiopathy^59^, we could state that selective blockade of 5-HT_2_ serotonergic receptors in T1D may be an useful therapeutic strategy to prevent or treat the alterations that initiate long-term cardiovascular complications.

Further studies will be required to determine whether sarpogrelate treatment may improve micro- and macrovascular disturbances derived from experimental long-term T1D, in order to confirm whether the selective 5-HT_2_ receptor blockade is a key therapeutic target in the treatment and/or prevention of cardiovascular complications resulting from chronic hyperglycaemia.

In conclusion, our findings suggest that blocking selectively 5-HT_2_ serotonergic receptor significantly improves endothelial function, alleviates the development of hypertension, renal hypertrophy and reduces oxidative stress. Therefore, this study supports that sarpogrelate treatment could establish an innovative therapeutic goal in the prevention and treatment of cardiovascular complications as consequence of diabetes.

## Methods

### Ethics in the study protocol

Housing conditions and experimental procedures were in accordance with regulations provided by the European Union on the use of animals for scientific purposes (2010/63/UE). This was enacted by Spanish legislation on 1^st^ February 2013 (R.D. 53/2013). All protocols were approved by the University of Salamanca Institutional Bioethics Committee (006N°201400037278).

### Compounds

The compounds utilized in the present study were: sarpogrelate hydrochloride was from ABBLIS Chemical LLC (Houston TX, US); ACh chloride, alloxan monohydrate, pentobarbital sodium, PE hydrochloride, indomethacin, L-NAME hydrochloride, SNP, thiobarbituric, tricloroacetic acid, N,N-dimethyl-9,9-biacridinium dinitrate (lucigenin), NADPH and ammonium diethyldithiocarbamate (DDC) were purchased from Sigma-Aldrich (Spain). TEA chloride was purchased from Tocris Bioscience (Bristol, UK). MDA bis-(dimethyl acetal) was purchased from Merk (Darmstadt, Germany). All other chemicals were of analytical grade. Stock solutions of the drugs were made up in ultrapure water, stored at −20 °C and appropriate dilutions were made on the day of the experiments.

### Animals

Twenty-four male Wistar rats (335 ± 10 g) were used in the present study. Rats were kept and supplied by the Animal House of the Faculty of Pharmacy of the University of Salamanca (PAE-SA001; Salamanca, Spain).

The animals were divided into three groups: normoglycaemic, diabetic and sarpogrelate-treated diabetic rats (n = 8 each). Diabetes was induced by a single injection of alloxan (150 mg/kg, s.c.) dissolved in saline solution. Rats were then maintained on tap water and regular food *ad libitum* for 28 days. Normoglycaemic rats received saline (1 ml/kg, s.c.), serving as controls. Non-fasting blood glucose levels, BW, HR and SBP were determined before and at 2, 7, 14, 21 and 28 days after alloxan (diabetic groups) or saline (control group) administration. Only rats with elevated blood glucose levels (>11 mM) were considered diabetic. Non-fasting blood glucose levels were determined by a glucometer (Accutrend Sensor^®^; Roche Diagnostics; Germany). SBP and HR were measured in awake rat using the tail-cuff method with a photoelectric sensor (NIPREM 546, Cibertec S.A, Madrid, Spain), where some determinations were made in each session for each animal, considering valid whether five consecutive measurements did not vary by 10 mmHg.

Sarpogrelate was administered dissolved in drinking water (30 mg/kg.day; p.o.), starting at 14 days of induction of T1D (i.e. during 14 days)^22^,^23^,^24^, in sarpogrelate-treated diabetic group.

At the end of the treatments (28 days), rats were anaesthetised with sodium pentobarbital (60 mg/kg, i.p.) and blood samples were collected. Then, thoracic aorta artery, heart and right kidney were extracted and placed in Krebs solution of the following composition (in mM): NaCl, 118; KCl, 4.7; CaCl_2_, 2.5; KH_2_PO_4_, 1.2; MgSO_4_•7H_2_O, 1.2; NaHCO_3_, 25 and glucose, 11 (pH = 7.4), and appropriately processed for further studies. Blood samples were centrifuged at 350 g for 10 min, at 4 °C, to obtain the plasma which was kept at −80 °C until use.

### Organs hypertrophy

The heart was removed and placed immediately in Krebs solution at 37 °C gassed with carbogen (5% CO_2_, 95% O_2_) to remove excess blood and subsequently kept at chilled Krebs. The atria were removed from the heart and all the epicardial fat was scraped off. The right and the left ventricle were separated, regarding the interventricular septum as an integral part of the left ventricle, and this portion was weighed. The LVH index was calculated using left ventricle weight/tibia length ratio (mg/mm).

The right kidney was dissected and fat separated. The RH index was calculated using kidney weight/tibia length ratio (mg/mm).

### Vascular reactivity

The thoracic aorta was carefully cleaned of fat and connective tissue and cut into rings (3 mm length) and placed in organ baths as we have indicated elsewhere by Kassan *et al*.^60^. The functional integrity of the endothelium was cheeked by assessing the relaxant response to ACh (10^−6^ M) in rings pre-contracted with PE (10^−6^ M). After a washout period, arteries were pre-contracted with PE (10^−6^ M) and at the steady maximal contraction, cumulative concentration-response curves to ACh (10^−8^ to 10^−4^ M) were performed in the absence and presence of pharmacological inhibitors: (1) NO-mediated relaxations: rings were incubated with the combination of indomethacin (10^−5^ M; a non-selective inhibitor of COX), plus TEA (10^−4^ M; a non-selective K^+^ channel blocker); (2) endothelium-dependent hyperpolarization-type relaxations: rings were incubated with indomethacin plus L-NAME (10^−4^ M; NO synthase inhibitor); and (3) prostacyclin-mediated relaxations: rings were incubated with L-NAME plus TEA. The preparations were incubated with the appropriate inhibitors for 30 min before the PE pre-contraction.

Endothelium-independent relaxation of the aortic arteries was assessed by pre-contracting with PE (10^−6^ M) followed by cumulative addition of SNP (10^−9^ to 10^−4^ M).

The responses to ACh and SNP are expressed as percentage of PE pre-contraction, and the responses to PE are represented as mg of contraction.

### Detection of vascular superoxide anion

O_2_•^−^ production was assessed by lucigenin-enhanced chemiluminescence assay. Briefly, segments of thoracic aorta were incubated in ROS Phosphate buffer (composition in mM: KH_2_PO_4_, 50; EGTA, 1 and Sucrose, 150, pH = 7.4) gassed with carbogen and maintained at 37 °C for 15 min. Then, samples were transferred into tubes containing ROS phosphate buffer supplemented with DDC (10 mM), NADPH (10^−4^ M) and lucigenin (5 μM). Lucigenin chemiluminescence was then recorded every 10 s for 5 min in a luminometer (LUMAT LB-9507, Berthold Technologies, Bad Wildbad, Germany). Production of O_2_•^−^ is expressed as relative luminescence units (RLU)/min/mg tissue.

### Lipid peroxidation measurement

Lipid peroxidation, a marker of oxidative stress, was determined by measuring the plasmatic MDA levels through the tiobarbituric acid reactive substances (TBARS) method, used by Kassan *et al*.^60^. Data are expressed as concentration of MDA, nmol/ml.

### Statistical procedures

All data are shown as mean ± SEM. Concentration–response curves were analysed using the GraphPad Prism 5.0 software (GraphPad, USA). Statistical analysis for significant differences between the different groups were performed with one-way analysis of variance (ANOVA) followed by the *post hoc* Bonferroni’s test. Significance was accepted at P < 0.05.

## Additional Information

**How to cite this article**: García-Pedraza, J.-Á. *et al*. Blocking 5-HT2 receptor restores cardiovascular disorders in type 1 experimental diabetes. *Sci. Rep*. **6**, 33979; doi: 10.1038/srep33979 (2016).

## Figures and Tables

**Table 1:** Monitored parameters in the different experimental groups.

	BW (g)	Glycaemia (mM)	SBP (mmHg)	HR (beats/min)
Control rats				
*Initial*	330.0 ± 5.0	6.0 ± 0.3	113 ± 3	353.0 ± 4.0
*Final*	390.4 ± 4.5	5.9 ± 0.2	115 ± 5	367.2 ± 3.8
Diabetic rats				
*Initial*	336.2 ± 4.5	6.3 ± 0.2	109 ± 3	343.3 ± 9.9
*Final*	334.0 ± 7.2^*^	26.4 ± 1.0^*^	142 ± 4^*^	367.2 ± 8.0
Sarpogrelate-treated diabetic rats				
*Initial*	338.0 ± 4.8	6.5 ± 0.2	114 ± 2	341.6 ± 9.8
*Final*	315.0 ± 10.9^*^	26.6 ± 1.1^*^	131 ± 1^*#^	352.4 ± 7.7

Initial and final (after 28 days) values of body weight (BW), glycaemia, systolic blood pressure (SBP) and heart rate (HR) in control, diabetic and sarpogrelate-treated diabetic rats (n = 8 each group). *P < 0.05 *vs* the corresponding value in control rats. ^#^P < 0.05 *vs* the corresponding value in diabetic rats. All values are expressed as mean ± SEM.
